# Clinical Outcomes in Carbapenem-Resistant Enterobacteriaceae Infections Treated With Ceftazidime-Avibactam: A Single-Center Observational Study

**DOI:** 10.7759/cureus.13081

**Published:** 2021-02-02

**Authors:** Balram Rathish, Arun Wilson, Anup Warrier, Shilpa Prakash, Rachana Babu, Sonya Joy

**Affiliations:** 1 Infectious Diseases, Aster Medcity, Kochi, IND; 2 Clinical Pharmacy, Aster Medcity, Kochi, IND; 3 Clinical Microbiology, Aster Medcity, Kochi, IND

**Keywords:** ceftazidime-avibactam, carbapenem resistant enterobacteriaceae, antimicrobial resistance, india

## Abstract

Introduction

Among the several newer beta lactam+beta lactase inhibitors (BL/BLI), ceftazidime-avibactam is the only drug showing activity against OXA-48-like producers. Hence, it is being increasingly used in India to treat infections caused by carbapenem-resistant Enterobacteriaceae (CRE), especially as a colistin-sparing agent. We have used ceftazidime-avibactam in patients suspected and confirmed to have CRE infections in our center, and present a retrospective analysis of our experience.

Methods

We conducted a single-center, retrospective study involving all patients who were treated with ceftazidime-avibactam for suspected and proven CRE infections during a one-year period at our 500-bedded hospital. Our primary objective for this study was taken as all-cause mortality. The secondary objectives were to determine the clinical cure, defined as the end of the treatment regimen with a resolution of primary infection and resistance to ceftazidime-avibactam in patients who underwent the Epsilometer test (E-test).

Results

A total of 103 patients who received ceftazidime-avibactam were identified. The all-cause mortality was 27% while a clinical cure was achieved in 73%. Fifty-two patients received empirical therapy and 51 patients received ceftazidime-avibactam for confirmed CRE infection. Forty-eight patients had an E-test done, out of which 79% of patients had CREs sensitive to ceftazidime-avibactam, and 21% of patients had ceftazidime-avibactam resistant CREs. A higher Sequential Organ Failure Assessment (SOFA) score, Charlson comorbidity index (CCI) score, intensive care unit (ICU) admission, inotrope requirement, and lower days of therapy (DOT) with ceftazidime-avibactam were found to be associated with increased mortality.

Conclusion

Colistin has been considered to be the last-line agent in CRE infections, but there are concerns about its adverse effects and the emergence of resistance. Given our relatively low mortality of 27% in CRE infections treated with ceftazidime-avibactam, coupled with the high susceptibility of the tested isolates, there may be a role for the empirical use of this drug in infections caused by CRE, especially in a setting where colistin may not be ideal.

## Introduction

Ceftazidime-avibactam is a relatively new, parenterally administered combination of third-generation cephalosporin ceftazidime and avibactam, a novel non-β-lactam β-lactamase inhibitor [1]. Combining ceftazidime with avibactam significantly (four to 1024 times minimum inhibitory concentration (MIC) reduction) improves its activity against most species of Enterobacteriaceae, as well as against Pseudomonas aeruginosa (approximately four times the MIC reduction) [2]. The combination of avibactam, however, does not increase the activity of ceftazidime against Acinetobacter species and most other anaerobic bacteria [2]. India reports high rates of multi-drug resistant bacteria with recent studies showing a high prevalence of carbapenem-resistant Enterobacteriaceae (CREs) with some studies reporting about three percent prevalence to other reports of up to 30% prevalence in Klebsiella pneumoniae and Escherichia coli [3-4]. Molecular analysis of the mechanism of resistance reports suggested a uniform distribution of the NDM and OXA-48 genes, with co-expression of NDM + OXA-48 in a small fraction of patients but no KPC or VIM [4].

Ceftazidime-avibactam has a broad spectrum of activity against Ambler molecular class A narrow-spectrum beta-lactamases, class A extended-spectrum beta-lactamases (ESBLs), and class A serine carbapenemases including KPC [5]. It also has activity against some class C such as KPC and class D carbapenemases, including OXA-48-like enzymes that may be present in Enterobacteriaceae. However, it has no activity against Ambler class B encompassing the metallo-beta-lactamases [5]. Among the several newer beta lactam+ beta lactase inhibitors (BL/BLI), ceftazidime-avibactam is the only drug showing activity against OXA-48-like producers [6]. Hence, ceftazidime-avibactam is being increasingly used in India to treat infections caused by CREs, especially as a colistin-sparing agent [7]. We have used ceftazidime-avibactam as both empirical and targeted therapy in patients suspected and confirmed to have CRE infections in our center, and present a retrospective analysis of our experience.

## Materials and methods

We conducted a single-center, retrospective study of all patients who were treated with ceftazidime-avibactam for a CRE infection from any source in a one-year period between September 1, 2019, and August 31, 2020, at our 500-bedded tertiary care hospital. The inclusion criteria for this study included patients aged more than 18 years who had received at least two days of therapy (DOT) of ceftazidime-avibactam in patients with renal dysfunction where dose modification was required, or 48 hours in patients without renal dysfunction. Pregnant women, children, and patients who received the drug for less than two DOT were excluded from the study. The patients were identified through our antimicrobial stewardship database. Baseline and demographic characteristics of the patients were accessed through electronic medical records and assessed from the time of the index infection. A full review was exempted by the institutional review board, as it was a retrospective study.

Syndromes of infections were defined using the Centers for Disease Control and Prevention/National Healthcare Safety Network Surveillance Definitions for Specific Types of Infections [8]. Baseline comorbidities of the patients were captured using the Charlson comorbidity index (CCI) [9]. The severity of the present infection was assessed using the Sequential Organ Failure Assessment (SOFA) score [10]. Both empirical and targeted therapy with ceftazidime-avibactam was included. Empirical therapy was given for cases with a high risk of CRE infections. Targeted therapy was initiated for confirmed CRE infections. Ceftazidime-avibactam was a restricted-use antibiotic in our center and the prescription of the agent was only done by authorized members of the antimicrobial stewardship team. Dosing was determined by the manufacturer's recommendation and the standard regimen for a CRE infection was taken as 2.5 grams thrice a day for 10 to 14 days. Concomitant therapy with antimicrobial spectrum against CRE infections was also recorded. The concomitant agent choice and dosing were prescriber specific.

Carbapenem resistance was measured using VITEK® 2 (BioMérieux, Marcy-l'Étoile, France) antimicrobial susceptibility (AST) cards for determining the MIC of the studied CREs against meropenem and ertapenem and confirmed with the disc diffusion method. Carbapenem resistance was defined as resistance to any carbapenem using current Clinical and Laboratory Standards Institute (CLSI) breakpoints [11]. Patients underwent AST of ceftazidime-avibactam, which was done using non-commercial Epsilometer tests (E-tests) based on availability at the time.

Our primary objective for this study was taken as all-cause mortality. The secondary objectives were to determine the clinical cure, defined as the end of the treatment regimen with a resolution of primary infection, and resistance to ceftazidime-avibactam in patients who underwent the E-test. Patient characteristics were described using descriptive statistics. Group comparisons were made using unpaired t-tests, Pearson’s test, or Fisher’s exact test. A p-value of <0.05 was considered significant.

## Results

During the study period, a total of 141 patients received ceftazidime-avibactam. Out of these, 103 patients fulfilled the pre-determined inclusion criteria. The all-cause mortality was 27% (n=28) while a clinical cure was achieved in 73% (n=75). The comparisons between outcomes are summarized in Table 1. The age of the patients ranged between 19 to 90 years of age with the mean (M), standard deviation (SD), median, and mode as 53.2 ± 17.3, 52, and 45 years, respectively. There was no significant association between ages in mortality groups (M=54.4, SD= 17.6) and clinical cure groups (M=52.7, SD=17.2) (t =0.4368, p=0.331). There were 78.5% (n=81) male patients and 21.5% (n=22) female patients. There was no significant association between sex and mortality (p= 0.596).

**Table 1:** Comparison of characteristics between the two outcome groups NA: Not Applicable, SOFA: Sequential Organ Failure Assessment, CCI: Charlson Comorbidity Index, DOT: Days of Therapy ^1 ^Expressed as mean ± standard deviation

Patient characteristics	Total (n=103)	Mortality (n=28)	Clinical cure (n=75)	p-value
Male sex	81	21	60	0.596
Age ^1^	53.2 ± 17.3	54.4 ± 17.6	52.7 ± 17.2	0.331
SOFA score ^1^	4.3 ± 3.2	5.7 ± 3.5	3.8 ± 2.9	0.004
CCI Score ^1^	4.1 ± 2.3	5 ± 2.4	3.8 ± 2.2	0.008
ICU admission	50	19	31	0.025
Inotrope requirement	30	15	15	0.001
Ventilatory requirement	46	16	30	0.127
Targeted therapy	51	10	41	0.120
Renal dose modification	26	8	18	0.803
Ceftazidime-avibactam DOT ^1^	8.1 ± 4.3	7 ± 3.8	8.5 ± 4.4	0.055
Monotherapy	69	20	49	0.642
Culture growth	63	19	44	0.497
E-test sensitive	38	10	28	1

The SOFA scores ranged between 0 to 16 with a mean, standard deviation, median, and mode scores of 4.3 ± 3.2, 4, and 2 respectively. The SOFA score in the mortality group (M=5.7, SD= 3.5) was significantly higher than that of the clinical cure group (M=3.8, SD=2.9) (t=2.69606, p=0.004). The CCI scores ranged between 0 and 10 with a mean, standard deviation, median, and mode scores of 4.1 ± 2.3, 4, and 3, respectively. The CCI score in the mortality group (M=5, SD=2.4) was significantly higher than in the clinical cure group (M=3.8, SD= 2.2) (t=3.18597, p=0.008). Among the studied patients, there was also a positive correlation between the SOFA and CCI scores (r=0.29, p=0.002).

Forty-eight point five percent (48.5%; n=50) patients required intensive care unit (ICU) admission. There was a significant positive association between ICU admission and mortality (p=0.025). Twenty-nine percent (29%; n=30) of patients were in shock, requiring inotropic support. There was a significant positive association between inotrope requirement and mortality (p=0.001). Forty-four point five percent (44.5%; n=46) patients required ventilatory support. There was no association identified between ventilatory requirement and mortality (p=0.127).

Fifty percent (50%; n=52) of patients received empirical ceftazidime-avibactam, that is, it was started before the isolation of an organism while 50% (n=51) patients had the drug started based on the microbiological culture identification of a CRE. There was no significant difference in mortality or clinical cure identified between empirical or targeted therapy (35% vs 20%) (p=0.120). Although 52 patients were empirically initiated on ceftazidime-avibactam, over the course of the study, 12 patients were subsequently proven to have a CRE infection with the isolation of the organism on culture. Thirty-eight percent (38%; n=40) of patients did not have a positive microbiological culture throughout the course of their illness.

Out of the 62% (n=63) patients with a positive microbiological culture, 48% (n=49) had an infection with K.pneumoniae, 6% (n=6) had E.coli, 4% (n=4) patients had P.aeruoginosa while 4% (n=4) had mixed bacterial growth. Clinically, 48% (n=50) had bacteremia, 16% (n=16) had pneumonia, 10% (n=11) had an intra-abdominal infection, 10% (n=11) had a central nervous system (CNS) infection, 9% (n=8) had a urinary tract infection (UTI), and 7% (n=7) had a skin and soft tissue infection (SSTI). There was no significant mortality difference identified between culture positive patients and culture negative patients (30% vs 22.5%) (p=0.497).

Twenty-five percent (25%; n=26) of patients had deranged renal functions and required renal dose modification of ceftazidime-avibactam. There was no association identified between renal dose modification or mortality (p=0.803). The DOT of ceftazidime-avibactam ranged between two to 22 days, with a mean, standard deviation, median, and mode of 8.1 ± 4.3, 7, and 7 days, respectively. There was no significant association between the day of initiation of ceftazidime-avibactam and mortality. Sixty-seven percent (67%; n=69) patients had monotherapy with ceftazidime-avibactam while 33% (n=34) had combination therapy with concomitant antibiotics with gram-negative coverage, including tigecycline, minocycline, polymixin, meropenem, levofloxacin, and aztreonam. There was no association identified between monotherapy or combination therapy with mortality (29% vs 23.5%) (p=0.642).

Forty-six percent (46%; n=48) patients had an E-test done, out of which 79% (n=38) patients had CREs sensitive to ceftazidime-avibactam and 21% (n=10) patients had ceftazidime-avibactam-resistant CREs. There was no association identified between E-test sensitivity and mortality (p=1).

## Discussion

South-Asia has become the hotbed of antimicrobial resistance, with some studies from India reporting a prevalence ranging from a modest 3% to an extremely high rate of around 65% CREs in clinical isolates [3-4,12-13]. Tigecycline and colistin are used as agents of choice for the treatment of CRE infections but treatment is complicated by uncertainties regarding the efficacy and adverse effects [14]. Newer agents like ceftazidime-avibactam have been increasingly used, as availability becomes wider with good clinical outcomes, however, data on clinical outcomes of ceftazidime-avibactam use in CREs is scarce from the Indian sub-continent. The mortality associated with CRE infections is higher than non-CRE infections but varies based on numerous factors [15-16]. Our study demonstrated overall mortality of 27%, which is similar to the 32% mortality reported in another similar multi-centric study from the USA on the use of ceftazidime-avibactam in CREs by King et al. [17]. A study by Shields et al. found 30-day mortality of 24% in CREs treated with ceftazidime-avibactam [18]. However, Alraddadi et al. found a 50% mortality in patients with CRE infections who received ceftazidime-avibactam from Saudi Arabia, highlighting the non-uniformity of mortality across geographical regions and patient characteristics [19].

A study on the clinical outcomes in patients receiving colistin by Prasannan et al., from our own center, found a mortality rate of around 10% in a sample of 150 patients who had received the drug [20]. However, they also found that almost 40% (n=59) of patients developed an acute kidney injury at some point during the colistin course. In our study, no patients were identified to have developed an AKI secondary to ceftazidime-avibactam. However, the other adverse drug events, including gastrointestinal manifestations, which are most commonly associated with ceftazidime-avibactam, were not available due to the retrospective nature of the study. Prasannan et al. only enrolled patients who had isolates susceptible to colistin and, hence, the resistance rates were not reported in the study, and the clinical details of patients who had colistin-resistant isolates were also not studied.

We also found significant positive associations between the initial SOFA scores and CCI scores of the patients and mortality. This is in keeping with previously published literature of worse outcomes in higher SOFA and higher CCI scores [9-10]. Mortality was significantly higher in patients who required ICU admissions and those who required inotropic support, which was in keeping with the high SOFA scores. However, we did not find an association between the requirement of ventilatory support and all-cause mortality. This could have been the result of unknown confounders or a result of an inadequate sample size.

In patients who underwent an E-test, almost 80% of CREs were sensitive to ceftazidime-avibactam. This was similar to the 73% reported in a large study by Spiliopoulou et al., but studies on resistance to ceftazidime-avibactam from clinical isolates were found to be few [21]. However, we failed to demonstrate a significantly different outcome in terms of mortality in patients with E-strip reported as sensitive, but this could have been as a result of the low number of patients who underwent the E-test. It could also have been due to a degree of sampling bias, where due to the limited availability of the E-test kits, only the most serious cases likely underwent the E-test, thereby further confounding the results.

Our study did have its limitations. We did not study the AST pattern of the isolates to colistin and other agents used for the treatment of CRE infections. Since this study was conducted retrospectively, parameters such as the SOFA score at the time of discharge or the adverse-effect profile of the drug could not be studied. Not all isolates underwent ceftazidime-avibactam AST due to the lack of a commercially available test. We were also not able to study the mechanism of resistance to ceftazidime-avibactam due to the widespread unavailability of molecular testing. The primary outcome chosen was all-cause mortality, and infection specific mortality could not be studied. There could also have been other confounders that were not accounted for.

## Conclusions

Colistin has been considered to be the last-line agent in CRE infections, but there are increasing concerns about its adverse effects, and the emergence of colistin resistance. Given our relatively low mortality of 27% in CRE infections treated with ceftazidime-avibactam, coupled with the relatively high susceptibility of the tested isolates to it, there may be a role for the empirical use of this drug in infections caused by CRE, especially in a setting of existing renal disease where colistin may not be ideal. We used ceftazidime-avibactam as a potential colistin-sparing agent in view of its relatively safer adverse-effect profile. The better adverse effect profile and similar outcomes associated with the use of ceftazidime-avibactam can support its use as the first-line therapy in CREs and accentuate its role as a colistin-sparing agent.
